# Clean air matters: an overview of traffic-related air pollution and pregnancy

**DOI:** 10.1590/S1518-8787.2017051006652

**Published:** 2017-02-08

**Authors:** Anne Dorothée Slovic, Carmen Simone Diniz, Helena Ribeiro

**Affiliations:** I Departamento de Saúde Ambiental. Faculdade de Saúde Pública. Universidade de São Paulo. São Paulo, SP, Brasil; II Departamento de Saúde, Ciclos de Vida e Sociedade. Faculdade de Saúde Pública. Universidade de São Paulo. São Paulo, SP, Brasil

**Keywords:** Pregnancy, Air Pollution, adverse effects, Maternal and Child Health, Environmental Health, Human Rights

## Abstract

The right to a healthy pregnancy and to giving birth in a safe environment is source of comprehensive research. Decent birth facilities, respect, and no discrimination are already recognized as fundamental rights, but an accurate look at the outdoor environment is required. Air pollution is a dangerous factor to pregnant women and newborns, many of whom highly exposed to traffic-related atmospheric pollutants in urban areas. Such exposure can lead to low birth weight and long-lasting effects, such as respiratory diseases and premature death. Thus, this commentary, based on the analysis of literature, presents the importance of the exposome concept and of epigenetics in identifying the role of the environment for better health conditions of pregnant women and newborns. In the final considerations, this study proposes the deepening of the subject and the mobilization in this regard, with a human rights-based approach to environmental health and to the increased awareness of pregnant women on the risks of air pollution and its effects on health.

## INTRODUCTION

Maternal health studies have shown the importance and the right of women to a safe and healthy pregnancy and delivery. Mistreatment during pregnancy, in particular during labor, has been associated with discrimination, abusive procedures^1,2^, and lack of communication of the multiple dimensions of intervention risk^3^, resulting in violation of women’s right to decent health-care during pregnancy and safe labor^4,5^. The World Health Organization (WHO) has acknowledged and emphasized women’s rights to assistance and respect during pregnancy and labor, calling for better practices, women’s empowerment, and participation in the decisions related to their body and to their children^a^. In addition, research has shown that the environment plays an important role in maternal health and birth outcomes, either via the mode of delivery on the baby’s immune system and health^1,6^ or by environmental degradation, such as air pollution. This said, there is still a gap in including the right to a healthy environment, in particular clean air, as a major component of a safe pregnancy and delivery. This commentary aims to highlight the issues related to air pollution, in particular in urban centers, and how it is essential to develop more exposure assessments to include the environment into a healthy pregnancy agenda.

## AIR POLLUTION AND MATERNAL HEALTH

Air pollution is defined by the WHO as “the contamination of the indoor or outdoor environment by any chemical, physical or biological agent that modifies the natural characteristics of the atmosphere”^7^and constitutes one of today’s major global environmental challenges. Furthermore, the WHO has recently published that air pollution is at the origin of seven million premature deaths, among which 3.7 million are attributed to outdoor air pollution^a^. The Organization for Economic Co-operation and Development (OECD) has projected that particulate matter, one of the major air pollutants, will be the second cause of premature deaths by 2050^b^.

In general, outdoor pollutants are generated by natural phenomena (e.g., volcanoes), industries (fixed sources), or internal combustion vehicles (mobile sources). In cities, atmospheric pollution is today one of the major environmental health issues affecting populations. It is of great importance to understand the overall effects of air pollution on human health. For instance, research has linked changes in life expectancy with changes in the levels of air pollution particulates in North American cities between the 1980s and 1990s^8^, when a decrease on particulate levels was associated with higher life expectancy. In urban areas, one of the most important primary sources of atmospheric pollution is car emission, posing a challenge because vehicle fleets are rapidly increasing^9^ and emission restrictions are sparse. Research has indicated that harm is bigger in regions where heavy traffic is concentrated and points to the consequences on the respiratory and circulatory systems^10-12^.

In addition to respiratory and cardiovascular diseases, birth outcomes, diabetes, and arteriosclerosis are also known to be associated with the exposure to atmospheric pollution. However, some populations carry a greater burden of disease^13-15^. This vulnerability is particularly found in children^10,16,17^ and older people^18-20^.

Increasing numbers of studies also correlate air pollution with women’s health and birth outcomes. Indeed, pregnant woman who are exposed to air pollution during the first trimester of pregnancy are more likely to have low weight or preterm newborns^14,21-23^. In a systematic review and meta-analysis of 60 studies^24^, a relation was found between pregnancy outcomes and particulate matter, nitrogen dioxide, and carbon dioxide; however, the study pointed out that the time and levels of exposure were major components to better understand consequences on health^24^. The exposure to air pollutants is today explored at the fetus stage via the mother, suggesting that reducing this exposure could be beneficial to the infant. Indeed, this early life exposure is linked to birth outcomes previously mentioned^25^. For pregnant women, some studies also suggest that air pollution is related to hypertension during pregnancy^26^ and pre-eclampsia during labor^27,28^ – one of the main causes of maternal and fetal death. Asthma is another important outcome of air pollution during pregnancy in exposed women, but also in children^10^, who are more likely to develop asthma in their early life.

### Research opportunities

Another approach for understanding the correlation between humans and environment is the exposome concept. This concept underlines the idea of adding all the different sorts of exposure in the life of a human being^29^. As stated by Wild^29^, this exposure implies considering general external factors as well as internal and specific internal ones. For instance, according to Wild^29^, “it is important to highlight the particular environment of the child, namely the body of its mother” (p. 24). In this concept, the overlapping of the different exposures is an essential component for assessing and measuring the exposure’s impact. One of the most useful tools to measure the exposome while understanding maternal health and air pollution are biomarkers (genetics among them) and sensor technologies such as air pollution monitoring stations. Epigenetic effects on prenatal and infant periods are well studied, but recent analyses have indicated the role of epigenetics using an exposome approach on labor or birth outcomes^30^. As explained by Dahlen et al.^30^, this research is promising in helping to understand how some procedures used during labor, such as oxytocin, and caesarean delivery can actually affect the epigenetic processes of mother and child.

The Helix project^31^, a European initiative, has carried on the objective to study pregnancy and early stages in life as a major component to understand lifetime consequences on health, considering this period as the “starting point for development of exposome” (p. 207).

## FINAL CONSIDERATIONS

As the link between maternal health and birth outcomes is of growing concern to a large body of researchers, some aspects remain of great importance to further analysis. The effects of air pollution on health are known, as well as the perspective of how problematic they can be to pregnant women and their babies. However, despite all the evidence, there is still no inclusion of the right to clean air and healthy environment as a major component of women’s right to a safe pregnancy and delivery; and some questions remain open to better understand what could be other measurements to evaluate these outcomes. In this sense, exposome and epigenetics may constitute an important field of study. Furthermore, as the human rights-based approach is integrated to health and environment, it will be essential for pregnant women to fully understand the environmental risks associated with unsafe pregnancy. As women are more empowered to understand the effects of air pollution during pregnancy and health outcomes, they will be able to make better choices. Scholars, environmentalists, policymakers, families, and health-care providers have a very important role in ensuring that environmental equity and a right-based approach are taken into account.

## Footnotes

a Organização Mundial da Saúde, Departamento de Saúde Reprodutiva e Pesquisa. Prevenção e eliminação de abusos, desrespeito e maus-tratos durante o parto em instituições de saúde. Genebra; 2014 [cited 2015 Jun 30]. Available from: http://apps.who.int/iris/bitstream/10665/134588/3/WHO_RHR_14.23_por.pdf

b Organization for Economic Co-Operation and Development. OECD environmental outlook to 2050: the consequences of inaction. Paris; 2012 [cited 2015 Sept 4]. Available from: http://www.oecd-ilibrary.org/environment/oecd-environmental-outlook-to-2050_9789264122246
